# Exploring the impact of different multi-level measures of physician communities in patient-centric care networks on healthcare outcomes: A multi-level regression approach

**DOI:** 10.1038/srep20222

**Published:** 2016-02-04

**Authors:** Shahadat Uddin

**Affiliations:** 1 Complex Systems Research Centre, University of Sydney, Darlington, New South Wales, Australia

## Abstract

A patient-centric care network can be defined as a network among a group of healthcare professionals who provide treatments to common patients. Various multi-level attributes of the members of this network have substantial influence to its perceived level of performance. In order to assess the impact different multi-level attributes of patient-centric care networks on healthcare outcomes, this study first captured patient-centric care networks for 85 hospitals using health insurance claim dataset. From these networks, this study then constructed physician collaboration networks based on the concept of patient-sharing network among physicians. A multi-level regression model was then developed to explore the impact of different attributes that are organised at two levels on hospitalisation cost and hospital length of stay. For Level-1 model, the average visit per physician significantly predicted both hospitalisation cost and hospital length of stay. The number of different physicians significantly predicted only the hospitalisation cost, which has significantly been moderated by age, gender and Comorbidity score of patients. All Level-1 findings showed significance variance across physician collaboration networks having different community structure and density. These findings could be utilised as a reflective measure by healthcare decision makers. Moreover, healthcare managers could consider them in developing effective healthcare environments.

A patient-centric approach to healthcare calls for increased collaboration among healthcare professionals who look after patients^1^,^2^. This leads to the development of an informal social network among healthcare professionals who collaborate while looking after patients. This informal network is known as patient-centric care network in the healthcare literature^3^. A patient-centric care network can therefore be thought as a group of healthcare professionals between whom collaborative connections or links emerge during the course of providing treatments to a common patient or a group of common patients. This study uses the multi-level regression approach to analyse and explore patient-centric care networks.

A multi-level regression model concerns the data that is structured in more than one hierarchical level. A sample from this data can be described as multistage data. First, a sample of higher level units is drawn (e.g. hospitals or organisations), and next a sample of available sub-units (e.g. patients or healthcare professionals in hospitals) is considered. In such data, individual observations at the lowest level are in general dependent on the all other available hierarchical levels, often called as explanatory variables, in the data. Separate linear regression models for each level are used to model the impact of the residuals from different hierarchical levels on the outcome variable in a multi-level regression model^4^. In this respect, multi-level regression models can be viewed as hierarchical systems of linear regression equations. A multi-level regression model is therefore considered as generalisations of linear regression models and is particularly appropriate for research designs where data for participants (e.g. patients) are organised at more than one level^4^.

There are numerous studies in the current literature exploring collaborations among healthcare professionals in a patient-centric care network. Mostly they examined hospital performance and patient outcomes by analysing collaboration networks among different participating members of patient-centric care networks, such as nurse-physician collaboration^5^, physician-pharmacist collaboration^6^, physician-patient collaboration^7^, hospital-physician collaboration^8^, and inter-professional and interdisciplinary collaboration^9^,^10^,^11^. After conducting an orderly review of studies of professionals’ network structures, Cunningham *et al*.^12^ noticed that cohesive and collaborative health professional networks can contribute to improving quality and safety of care. In a quasi-experiment on general medicine patients where experimental group received care from a specially designed care management plan that facilitated higher collaboration among hospital staff and control group received the usual care, Cowan *et al*.^13^ noticed that average hospital length of stay, total hospitalisation cost and hospital readmission rate were significantly lower for patients in the experimental group than the control group. Sommers *et al*.^14^ examined the impact of an interdisciplinary and collaborative patient-centric practice intervention involving a primary care physician, a nurse and a social worker for community-dwelling seniors with chronic illnesses. The intervention group received care from their primary care physician working with a registered nurse and a social worker, while the control group received as usual care from primary care physicians. They noticed that the intervention group produced better results in relation to readmission rates and average office visits to all physicians. From this brief review of the present healthcare literature, it is clearly evident that enormous research effort has been given to explore and analyse patient-centric care networks. However, none of these studies attempted to explore patient-centric care networks from a hierarchical point of view of the available healthcare data. By following a multi-level regression model, this study examines the impact of the structure of patient-centric care networks on patient outcomes.

## Methods

### Research framework for multi-level analysis of patient-centric care network

This study considered patient-centric care networks that are being emerged among different healthcare professionals during the hospitalisation period of patients. From the point of view of patients, they receive different healthcare services during their hospitalisation periods. After some period, based on the suggestions of hospital physicians, they are discharged only if their health conditions improve. Patients have different socio-demographic characteristics and could show different level of responses to the prescribed medications by hospital physicians. In return, patients who have private health insurance pay bills to their corresponding health insurance organisations according to their membership conditions. Government agencies (e.g. Medicare in Australia) pay the bill for patients who do not have any private health insurance.

**Figure 1:**
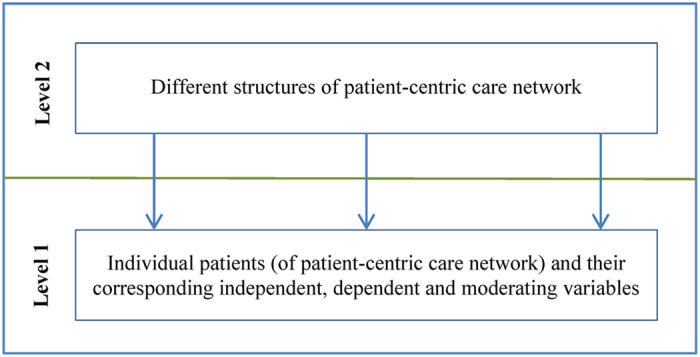
Conceptual multi-level regression models for patient-centric care network.

**Figure 2:**
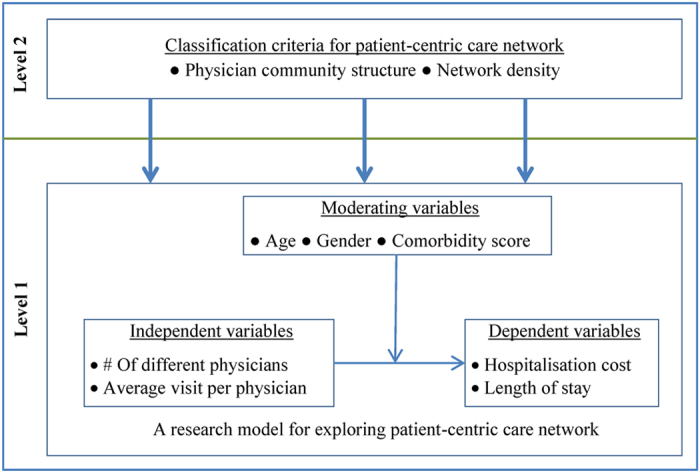
A multi-level regression model for analysing patient-centric care network.

From a higher hierarchical point of view (i.e. hospitals’ point of view), different hospitals could have same or different organisational practice culture and policy. This defines the way newly admitted patients will be treated. Furthermore, this difference in organisational practice culture and policy guides the development of collaboration network among healthcare professionals during the course of providing care to hospitalised patients^15^. The structure of this collaboration network affects, in addition to the patient-level factors (e.g. disease severity and socio-demographic characteristics), the patient-level healthcare outcomes (e.g. total hospitalisation cost and hospital length of stay)^16^. This study is particularly interested to explore the impact of the collaboration network among physicians on the patients’ healthcare outcomes. By considering these two hierarchical viewpoints, a conceptual multi-level framework for exploring patient-centric care network is illustrated in Fig. 1. In this framework, patient-level information is utilised at the first level and structural information of patient-centric care network is used at the second level. By considering different patient-level (i.e. Age, Gender, Hospitalisation cost, Length of stay, Comorbidity score, Number of different physicians visited a patient and Average visit per physician) and hospital-level (i.e. Community structure and Network density of physician collaboration network) measures, an instantiation of the conceptual multi-level framework of Fig. 1 is presented in Fig. 2.

### Research data source

This study utilised computerised (as well as de-identified) electronic health insurance claim dataset which was provided by an Australian not-for-profit health insurance organisation and contained physician-patient interaction information over a period of five years. The usage of this electronic health insurance claim dataset for research purpose has been approved by the University of Sydney’s ethics committee and the ethics committee of the corresponding health insurance organisation. This dataset included electronic health insurance claim details of 2,352 hip replacement patients who received health services from 2,229 physicians in 85 different hospitals. There are several advantages of using administrative health insurance claim data for research purpose. First, insurance claim databases usually tend to be highly representative of a large population. This permits enhanced precision and study rare events. Second, data analysis is inexpensive as the data are already collected and computerised. Third, insurance claim data are free from selection and response bias. Finally, claim data preclude any imposition on patient, physicians or other providers.

An admission of a patient in a hospital generates many physician and hospital claims submitted to the corresponding health insurance provider. Physician claims render details of services that had been provided by physicians during their visits to hospitalised patients. On the other hand, hospital claims provide details of services (e.g. imaging and pathology) provided by other hospital staff and other admission-related information (e.g. admission date, length of stay, and patient personal information including date of birth and gender). This study mostly utilised details of physician and hospital claims for extracting and quantifying required measures that are under consideration as in Fig. 2. The basic statistics about these measures is given in Table 1.

### Construction of physician collaboration networks

**Figure 3:**
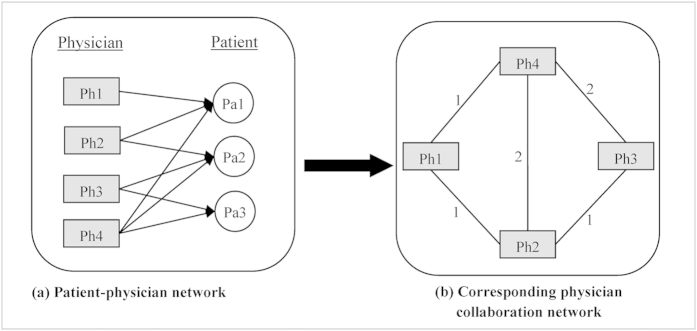
Construction of a physician collaboration network. In a hospital (say *H1*), patient *Pa1* is visited by *Ph1*, *Ph2* and *Ph4* physicians, patient *Pa2* is visited by *Ph2*, *Ph3* and *Ph4* physicians, and physician *Ph3* and *Ph4* visit patient *Pa3*. This *patient-physician* network is depicted in the panel (**a**). The corresponding physician collaboration network for this *patient-physician* network is demonstrated in the panel (**b**). In this physician collaboration network, there are network connections with weight 1 between *Ph1* and *Ph2*, between *Ph1* and *Ph4*, and between *Ph2* and *Ph3* because they visited a common patient. The weight of the links between *Ph2* and *Ph4* and between *Ph3* and *Ph4* are two as they visited two common patients.

In a patient-centric care network, two or more physicians usually collaborate in treating the corresponding patient. Presence of common patients in different patient-centric care networks among a group of physicians therefore renders to the creation of a physician collaboration network. A group of physicians belong to a physician collaboration network if any physician of that group has at least a common patient with one or more of the remaining group members. Physician collaboration can therefore be thought as a ‘patient-sharing network among physicians’. From physician claims, this study identified physicians who visited a common hospitalised patient. From this information, this study mapped physician collaboration network. If two physicians visited a common hospitalised patient then this study assigned a link of weight one between them. Similarly, if two physicians visited two common hospitalised patients then the assigned link between them will have a weight of two and so on. There are studies found in the healthcare literature that followed approaches similar to this approach for constructing collaboration networks among healthcare professionals^17^,^18^. An illustration of the construction of such a physician collaboration network is presented in Fig. 3. This study constructed 85 different physician collaboration networks from physicians’ patient-sharing network for 85 hospitals. These physician collaboration networks do not show much difference in terms of different network properties. In respect of the degree distribution, for instance, almost every networks show similar property– have few network hubs (i.e. actors with very high number of links with other actors) along with many actors having low links. Moreover, a positive correlation has been noticed between the number of communities and network size for these networks. On the other side, a negative correlation has been found between the number of communities and network density.

### Level-2 variables (Network measures)

In a multi-level model, level-2 variables are utilised only for grouping purpose. This study considered two network measures as level-2 variables in the proposed multi-level regression model: Community structure and Network density. The selection of these grouping variables is based on a network theory which is the theory of centralisation, proposed by Bavelas^19^. As stated in this theory, group performance in a collaborative environment where individuals work towards achieving a common goal depends on the structure of communication patterns among the members of that group. Since these two variables are continuous variables by their nature, this study followed a statistical approach to convert them into categorical variables.

### Community structure within physician collaboration networks

**Figure 4:**
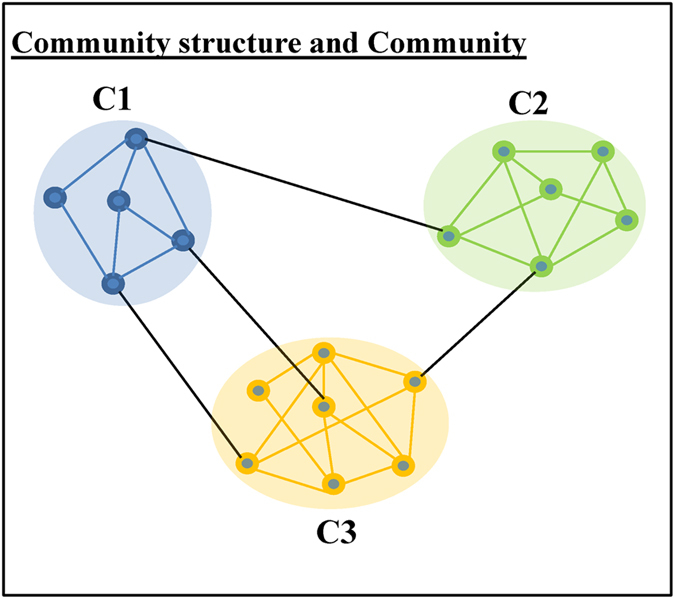
Illustration of network communities in an abstract social network. A network is said to have ‘community structure’ if the nodes of that network can easily be divided into sets of groups such that each set of nodes is densely connected internally. Each set of nodes is called a ‘community’. In the above network, there are three communities (i.e. C1, C2 and C3). Any node of these communities has more links with other nodes of the same community compared to the number of links with other nodes from other communities.

**Figure 5:**
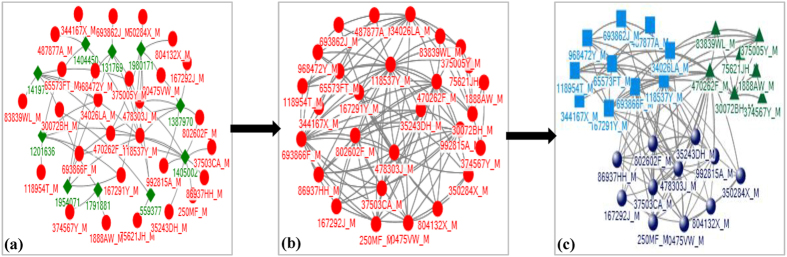
An example of the construction of a representative physician collaboration network and extraction of its communities from the research dataset of this study. In this physician collaboration network, 28 physicians visited 57 times to 10 patients. The label for each actor represents a system generated unique ID for each actor. Each physician ID ends with the suffix of “_M” and each physician ID consists of only numbers. (**a**) Physician-patient links – a red circle represents a physician and a patient is being represented by a green diamond; (**b**) Corresponding physician collaboration network; and (**c**) Detected communities within the physician collaboration network–physicians belonging to the same community are being represented by the same shape with the same colour. Three communities have been identified in this representative physician collaboration network.

Detecting communities helps us to make sense about any given network. Communities consist of entities, called nodes, and their relationships, called edges. They emerge as dense parts in a network while they may have a few relationships to each other. An illustrative example of network communities in an abstract social network is presented in Fig. 4. In order to uncover the community structure of each physician collaboration network, this study applied an algorithm introduced by Amiri *et al*.^20^. Inspired by the original firefly algorithm, this algorithm is based on a multi-objective optimisation approach. There are many community detection algorithms in the literature that were inspired by the behaviour of social insects called fireflies^21^,^22^. Since communities are highly connected internally and sparsely connected externally, community detection problems can be formulated with two different objectives: maximisation of internal links and minimisation of external links^23^. By defining a tradeoff between these two objectives and considering original firefly algorithm, Amiri *et al*.^20^ proposed an enhanced algorithm for community detection. Compared to other community detection algorithms available in the present literature^24^,^25^,^26^, this algorithm showed higher efficiency at discovering community structures of complex networks when implemented and tested on several real world and synthetic datasets^20^. The weak point of this algorithm is its inability for discovering overlapping communities. However, this study does not need to consider such a community detection algorithm that can detect overlapping communities since it did not consider any physician attribute in detecting communities within physician collaboration networks. Therefore, the selection of the above community detection algorithm will not affect the desired level of efficiency in detecting communities within physician collaboration networks. An illustration of the construction of a representative physician collaboration network and detection of its communities from the research dataset of this study has been presented in Fig. 5.

Three factors affect the number of communities produced by enhanced firefly algorithm proposed by Amiri *et al*.^20^: the number of completely disconnected nodes in the network; the degree distribution; and the size of the network. There is no disconnected node in any of the 85 physician collaboration networks of this study. This is because physician collaboration network were constructed on the concept of ‘patient-sharing network among physicians’, as described in the previous section. On the other hand, it is noticed that there is no significant difference among the degree distributions of all physician collaboration networks. For normalising the impact of network size, this study therefore considered the average number of nodes (i.e. physicians) per community to capture community structure in different physician collaboration networks. For example, if there are 4 communities found in a network which has 20 nodes then the average number of nodes per community for that network is 5 (i.e. 20 ÷ 4 = 5). In order to convert this level-2 variable into a categorical variable this study first calculated the range and standard deviation (∂) of this variable (i.e. average number of nodes per community) for all 85 physician collaboration networks. Based on these two statistical measures, this study then defined five categories as reported in Table 2.

### Network density

This is the second level-2 variable of the proposed multi-level regression model for analysing patient-centric care networks. Network density represents the number of links of a network as a ratio of the number of all possible links among all nodes of that network and can be calculated by the following equation^27^:


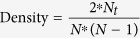


Where, *N*_*t*_ is the number of link in a network and *N* is the number of nodes of that network. For converting density values of 85 physician collaboration networks, this study followed same approach as it followed for the first level 2 variable (i.e. the number of physicians in a community). The detail of different categories for network density is also reported in Table 2.

### Level-1 variables (Non-network measures)

All level-1 variables considered in this study are based on different measures of individual hospitalised patient. As illustrated in Fig. 2, this study considered three different types of level-1 variable: independent variable; dependent variable; and moderating variable.

### Independent variables

This study considered two independent variables: Number of different physicians and Average visit per physician. For a patient, the number of different physicians represents how many different physicians visited that patient during her hospitalisation period. The second independent variable is the average visit per physician which represents the number of visits, on average, a physician made to a particular hospitalised patient. For instance, if a patient had been visited 20 times by 5 different physicians during her hospitalisation period then then the number of different physicians (i.e. the first independent variable) is 5 and the average visit per physician (i.e. the second independent variable) is 4 (i.e. 20 ÷ 5 = 4).

### Dependent variables

The first dependent variable is the hospitalisation cost which was calculated, for a hospitalised patient, by summing up cost for all services including hospital accommodation cost, physicians’ visit fees and cost for any medical test provided to that patient. Another dependent variable is the length of stay which represents the total number of days that a patient stayed at hospital for a hospital admission. The usability of these two indices as healthcare outcome measures can be found extensively in the present healthcare literature^28^,^29^,^30^.

### Moderating variables

Three variables were considered as moderating variables: two socio-demographic variables (i.e. Age and Gender) and a comorbidity variable (i.e. Charlson-Deyo index). For a hospitalised patient, the first two moderating variables have explicit meanings that can be easily distinguished by their names. Charlson-Deyo index, which was described by Charlson *et al*.^31^ and latter adapted for use with International Classification of Disease (ICD) by Deyo *et al*.^32^, is a comorbidity risk adjustment method that has been utilised widely with administrative data in the literature. Since the research dataset of this study was considered from an Australian health insurance organisation, this study considered a variation of this comorbid index developed by Sundararajan *et al*.^33^ for Australian standard.

## Results

This study followed the guidelines described by Field^4^ and Snijders^34^ for compiling the multi-level regression model as presented in Fig. 2. Since there are two dependent variables (i.e. Hospitalisation cost and Length of stay) at Level-1 and two grouping variable at Level-2 (i.e. Community structure and Network density), this study developed four multi-level regression models. For the ease of presentation, these four models have been presented in two tables, Table 3(a,b), separating them based on two dependent variables (i.e. Hospitalisation cost and Length of stay) respectively.

The process to test a two-level regression model is as follows: first without indicating the hierarchical variables (i.e. Level-2 variables), the impact of independent and moderating variables of Level-1 on the outcome variables of the same level needs to be checked. This step is similar to the steps followed in a multiple regression model. Second, the clustering variables (i.e. grouping variables or Level-2 hierarchical variables) will be considered to investigate: (i) how these groupings will affect the *estimate* (i.e. co-efficient *b*_*i*_) and *p-value* of the relations among Level-1 variables; and (ii) whether inclusion of these grouping variables has made any difference to the initial model as described in the first step. This difference can be tested by observing the change in *−2LL* (i.e. −2*Log Likelihood)^4^.

Table 3 reports the impact of independent (i.e. Number of different physicians and Average visit per physician) and moderating variables (i.e. Age, Gender and Comorbidity score) of Level-1 on the two dependent variables (i.e. Hospitalisation cost and Length of stay) of the same level by considering: (i) first absence; and (ii) then presence of two clustering variables (i.e. Community structure and Network density) of Level-2. The *estimate* and *p-value* of the impact of eight regression parameters (i.e. independent and moderating variables) on the hospitalisation cost are illustrated in Table 3(a). Although these *estimates* and *p-value* vary over three conditions (i.e. absence and presence of Level-2 grouping variables: do not consider grouping variable, consider Community structure and consider Network density), the first five parameters always show significant impact (*p < 0.05*) on the hospitalisation cost (see corresponding cells of column 3, column 5 and column 7 of Table 3(a) for the first five parameters). The three moderating variables (i.e. Age, Gender and Comorbidity score) do not moderate the relation between the average visit per physician and hospitalisation cost (see corresponding cells of column 3, column 5 and column 7 of Table 3(a) for the last three parameters). By considering the absence and presence of Level-2 grouping variables, Table 3(b) presents *estimate* and *p-value* of the impact of eight regression parameters on the length of stay. These *estimates* and *p-values* vary over three conditions (i.e. absence and presence of Level-2 grouping variables: do not consider grouping variable, consider Community structure and consider Network density). However, three of these parameters (i.e. Average visit per physicians, Number of different physicians*Age and Average visit per physicians*Gender) always show significant impact (*p < 0.05*) on the length of stay. The first independent variable (i.e. Number of different physicians) does not show any significant impact on the length of stay (*p* = *0*.*689* from the second row of Table 3(b)) although it shows significant impact on the hospitalisation cost (*p* = *0.000* from the second row of Table 3(a)).

In order to assess whether consideration of grouping variables has made any difference to the model, it is required to investigate the change in *−2LL* (i.e. −2*Log Likelihood) which is a reliable measure to check significance of changes to a model^4^. A grouping variable (i.e. Level-2 variable) will show significant impact when its inclusion will change the −*2LL* significantly^4^. Table 4 presents different values for −*2LL* and their change statistics for the four multi-level regression models that are based on two dependent and two grouping variables from Level-1 and Level-2, respectively. As noticed in the last column of Table 4, both grouping variables (i.e. Community structure and Network density) have made a significant difference to two initial models (one for the Hospitalisation cost and another for the Length of stay). For example, for the first dependent variable (i.e. Hospitalisation cost) inclusion of the first grouping variable (i.e. Community structure) changed the χ^2^ value by 11.20 (i.e. 43338.85 − 43327.65 as in the second row of Table 4), which is greater than 6.63 (i.e. *p = 0.01* for *df *= 1).

A falsification test has been carried out in order to check the robustness of the above mentioned findings of this study. The structure of 17 (i.e. 20%) patient-centric care networks has been changed by deleting all links first and then assigning the same number of links randomly between patients and physicians of the corresponding patient-centric care networks. Then the relations between any pair of independent variables (i.e. Number of different physicians and Average visit per physician) and dependent variables (i.e. Hospitalisation cost and Length of stay) have been checked again. It is noticed that *estimates* and corresponding *p-values* have been changed by a considerable amount in each of the possible four combinations between two independent variables and two dependent variables, leading to the change of the original findings of this study. For example, after changing the structures of 17 patient-centric care networks it is found that the *estimate* and *p-value* between the number of different physicians and length of stay are 1.57 and 0.045 without the presence of any grouping variable, whereas they were 0.09 and 0.689, respectively, before. This falsification test therefore confirms the robustness of the findings of this study.

## Discussion and Conclusion

In a patient-centric care network, the average visit per physician significantly predicted hospitalisation cost and hospital length of stay. However, the number of different physicians predicted only hospitalisation cost significantly. For some combinations of independent and dependent variables (e.g. Number of different physicians and Hospitalisation cost), all moderating variables showed significant moderating impact while for some others (e.g. Average visit per physician and Hospitalisation cost) they did not. The impact of independent and moderating variables on dependent variables showed significant variance across patient-centric care networks having different level of community structure and network density.

For the level-1 model, hospitalisation cost and length of stay can significantly be predicted by the average visit per physician. Higher number of physicians making a specific number visits to a hospitalised patient is better, in terms of lower hospitalisation cost and length of stay, compared to the situation whether smaller number of physicians make the same number of visits to that patient. There are dependencies between physicians’ visits to a hospitalised patient. For instance, before having a hip replacement surgery by a specialist physician a patient needs to be visited by an anesthetic physician. For some reasons, if the anesthetist makes a delay then the specialist physician will also need to wait. An increased number of physician visits to a hospitalised patient will make this type of delay highly probable to occur, which could be either a cause or an effect of increased healthcare utilisation. Patient age showed significant moderating impact in all cases for the relation between the number of different physicians and both dependent variables. The underlying reason for this finding could be the fact that surgical complexity for hip replacement patients, who were the research subject of this study, increases with older patients^35^.

The grouping parameters (i.e. Community structure and Network density) have significant impact on the model described in the first level of Fig. 2 across different patient-centric care networks. Bavelas theory of centralisation^19^ can provide a possible explanation for this association. According to this theory, the communication pattern among a group of individuals has significant impact on performance of that group in a collaborative and goal-oriented environment. Different communication patterns provide different level of knowledge sharing opportunity among individuals. Physicians have different level of communication patterns among themselves in their respective physician collaboration networks. Some physician collaboration networks are segmented into higher number of communities. In addition to having a smaller community size on average, physicians are clustered into small groups where they are strongly connected internally in those networks. On the other hand, some other physician collaboration networks are segmented into lower number of communities. The community size is higher in those physician collaboration networks. In respect to the network density, some physician collaboration networks are dense compared to others. In a dense physician collaboration network, each member physician is easily reachable by her colleagues compared to a sparse physician collaboration network. This variability in community census and network density across different physician collaboration networks facilitates different level of knowledge sharing opportunity among physicians, which is very critical to the success and survival in competitive environments for hospital organisations^36^.

By following the multi-level regression approach, this study showed significant variance in the relation among different patient-related measures across different physician collaboration networks having various community structures and network density values. This study is subject to several considerable limitations. First, based on the presence of shared patients this study constructed patient-centric care networks and physician collaboration networks from health insurance claim data. However, it cannot be known what information, if any, pass across the ties defined by shared patients. Second, the research data included only total hip replacement patients. Thus, future analyses are required for patients suffering from other illnesses such as knee surgery or brain cancer in order to claim the generality of the findings of this study. Third, this study considered only quantitative measures (i.e. Hospitalisation cost and Length of stay) as outcome variables. It did not consider any qualitative measure although there are many qualitative measures (e.g. patient satisfaction^37^) that have been used as outcome measures in the healthcare research. Finally, this study did not consider the change in team memberships (e.g. how long the same group of physicians providing care to different patients, or how frequently team membership changed over the time) and their impacts (e.g. whether, or not, the same team provides better care to different patients) on healthcare outcome.

## Additional Information

**How to cite this article**: Uddin, S. Exploring the impact of different multi-level measures of physician communities in patient-centric care networks on healthcare outcomes: A multi-level regression approach. *Sci. Rep*. **6**, 20222; doi: 10.1038/srep20222 (2016).

## Figures and Tables

**Table 1:** Basic statistics of measures of the proposed framework.

Item	Total	Average	Range	Standard deviation (∂)
Number of patients	2352	–	–	–
Male	1128	–	–	–
Female	1224	–	–	–
Number of physicians	2229	–	–	–
Number of hospital	85	–	–	–
Patient age (years)	–	69.09	40.10 to 99.36	12.09
Hospitalisation cost ($AUD)	–	31036	3073 to 178247	21315
Length of stay (days)	–	10.79	2 to 119	10.05
Average number of physicians per community (for capturing Community structure)	–	13.42	3.20 to 37.80	6.96
Network density (for capturing Network density)	–	0.27	0.08 to 0.63	0.11

**Table 2:** Categorical information for two grouping variables (i.e. Level-2 variables).

Category name and criteria	Grouping variables from the second level			
	Community structure^a^		Network density	
	*Range*	*Distribution*^b^	*Range*	*Distribution*^b^
Category A (Min, Min+∂)	3.20 to 10.16	29 (34.12%)	0.08 to 0.17	11 (12.94%)
Category B (>Min+∂, Min+2*∂)	>10.16 to 17.12	36 (42.35%)	>0.17 to 0.28	40 (47.06%)
Category C (>Min+2*∂, Min+3*∂)	>17.12 to 24.08	12 (14.12%)	>0.28 to 0.39	20 (23.53%)
Category D (>Min+3*∂, Min+4*∂)	>24.08 to 31.04	6 (7.06%)	>0.39 to 0.50	11 (12.94%)
Category E (>Min+4*∂, Max)	>31.04 to 37.80	2 (2.35%)	>0.50 to 0.63	3 (3.53%)

^a^Captured by average # of physicians per community.

^b^Indicates how many of 85 physician collaboration networks belong to this category.

**Table 3:** Impact of independent and moderating variable on the dependent variables by considering first absence and then presence of grouping or clustering variables of the second level.

(a) For hospitalisation cost									
Parameters	Hospitalisation cost								
	Do not consider grouping variables of Level-2			Consider *Community structure* of Level-2			Consider *Network density* of Level-2		
	Estimate	F-ratio	p-value	Estimate	F-ratio	p-value	Estimate	F-ratio	p-value
# of different physicians	5788.11	276.77	0.000	5867.60	285.21	0.000	5722.59	276.65	0.000
Avg. visit per physician	3369.98	24.36	0.000	3138.29	20.89	0.000	3368.67	24.39	0.000
# of different physicians*Age	−31.88	48.34	0.000	−32.98	51.87	0.000	−31.87	48.59	0.000
# of different physicians*Gender	283.51	5.88	0.015	280.81	5.82	0.016	300.18	6.67	0.010
# of different physicians*Comorbidity score	−1004.62	4.39	0.036	−1075.95	5.07	0.024	−1022.81	4.61	0.032
Avg. visit per physician*Age	−16.71	3.68	0.055	−14.10	2.60	0.107	−16.39	3.55	0.060
Avg. visit per physician*Gender	−329.99	2.52	0.113	−309.47	2.23	0.136	−359.03	3.01	0.083
Avg. visit per physician*Comorbidity score	702.13	0.74	0.389	805.39	0.98	0.322	809.39	0.79	0.376

(b) For length of stay									
Parameters	Length of stay								
	Do not consider grouping variables of Level-2			Consider *Community structure* of Level-2			Consider *Network density* of Level-2		
	Estimate	F-ratio	p-value	Estimate	F-ratio	p-value	Estimate	F-ratio	p-value
# of different physicians	0.09	0.16	0.689	0.07	0.08	0.779	0.04	0.02	0.878
Avg. visit per physician	1.12	5.56	0.019	1.21	6.38	0.012	1.28	7.26	0.007
# of different physicians*Age	0.01	7.86	0.005	0.01	8.55	0.003	0.01	9.37	0.002
# of different physicians*Gender	−0.10	1.49	0.223	−0.10	1.44	0.231	−0.10	1.36	0.244
# of different physicians*Comorbidity score	0.26	0.62	0.431	0.29	0.74	0.391	0.28	0.71	0.400
Avg. visit per physician*Age	0.01	1.29	0.255	0.01	0.94	0.332	0.01	0.65	0.419
Avg. visit per physician*Gender	−0.34	5.39	0.020	−0.34	5.58	0.018	−0.36	6.07	0.014
Avg. visit per physician*Comorbidity score	−0.18	0.10	0.750	−0.22	0.15	0.704	−0.55	0.13	0.719

Total number of observations at level-1 and at level-2 is 2,352 and 85, respectively.

Total number of observations at level-1 and at level-2 is 2,352 and 85, respectively.

**Table 4:** Different values of *–2LL* and their statistics for checking the impact of grouping variables (i.e. Level-2 variables).

Dependent variable (Level-1)	Group variable (Level-2)	–2LL	Change of –2LL ( 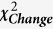 )	Significance^a^
Hospitalisation cost	None	43338.85	11.20	*p* < *0.01*
	Community structure	43327.65		
Hospitalisation cost	None	43338.85	18.24	*p* < *0.01*
	Network density	43320.61		
Length of stay	None	14065.37	10.56	*p* < *0.01*
	Community structure	14054.81		
Length of stay	None	14055.37	9.59	*p* < *0.01*
	Network density	14045.78		

^a^
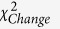
= 6.63 at p = 0.01 and df = 1.
